# 18β-Glycyrrhetinic Acid Induces Metabolic Changes and Reduces *Staphylococcus aureus* Bacterial Cell-to-Cell Interactions

**DOI:** 10.3390/antibiotics11060781

**Published:** 2022-06-08

**Authors:** Alan J. Weaver, Timothy R. Borgogna, Galen O’Shea-Stone, Tami R. Peters, Valérie Copié, Jovanka Voyich, Martin Teintze

**Affiliations:** 1 Department of Chemistry & Biochemistry, Montana State University, Bozeman, MT 59717, USA; alan.weaverjr@gmail.com (A.J.W.J.); galen.osheastone@student.montana.edu (G.O.-S.); tami.peters@montana.edu (T.R.P.); 2 Department of Microbiology & Cell Biology, Montana State University, Bozeman, MT 59717, USA; timothy.borgogna@montana.edu

**Keywords:** *Staphylococcus aureus*, antibacterials, antibiotics, resistance, staphyloxanthin, nuclear magnetic resonance, metabolomics, MRSA, planktonic cell cultures, biofilm infections

## Abstract

The rise in bacterial resistance to common antibiotics has raised an increased need for alternative treatment strategies. The natural antibacterial product, 18β-glycyrrhetinic acid (GRA) has shown efficacy against community-associated methicillin-resistant *Staphylococcus aureus* (MRSA), although its interactions against planktonic and biofilm modes of growth remain poorly understood. This investigation utilized biochemical and metabolic approaches to further elucidate the effects of GRA on MRSA. Prolonged exposure of planktonic MRSA cell cultures to GRA resulted in increased production of staphyloxanthin, a pigment known to exhibit antioxidant and membrane-stabilizing functions. Then, 1D ^1^H NMR analyses of intracellular metabolite extracts from MRSA treated with GRA revealed significant changes in intracellular polar metabolite profiles, including increased levels of succinate and citrate, and significant reductions in several amino acids, including branch chain amino acids. These changes reflect the MRSA response to GRA exposure, including potentially altering its membrane composition, which consumes branched chain amino acids and leads to significant energy expenditure. Although GRA itself had no significant effect of biofilm viability, it seems to be an effective biofilm disruptor. This may be related to interference with cell–cell aggregation, as treatment of planktonic MRSA cultures with GRA leads to a significant reduction in micro-aggregation. The dispersive nature of GRA on MRSA biofilms may prove valuable for treatment of such infections and could be used to increase susceptibility to complementary antibiotic therapeutics.

## 1. Introduction

The rapid development of bacterial resistance to most used antibiotics requires a renewed effort to generate new antibacterial compounds that could be used to develop more effective alternative treatment strategies, particularly as it relates to biofilm infections. Natural products and their derivatives have been the source of most antibiotics and continue to provide a rich source of novel compounds [1,2]. One plant species contributing significantly to drug discovery is *Glycyrrhiza* spp., which are members of the licorice family [3,4].

Licorice roots have a long history of use in Chinese herbal medicine [5,6]. The fractionation of the *Glycyrrhiza* spp. roots has led to the discovery of numerous compounds with activity against a wide variety of ailments and diseases, including use as antimicrobials [3,4,5]. The bioactivity of these roots has been attributed to their triterpene saponins and flavonoid contents [4]. Among these bioactive compounds is glycyrrhizic acid, which, following ingestion, is converted by commensal gut bacteria to 18β-glycyrrhetinic acid (GRA), a pentacyclic triterpene known to exhibit efficacy against diverse ailments, including bacterial infection [3,7,8].

*S. aureus* is a Gram-positive, opportunistic bacterium that colonizes the skin of healthy individuals, but can also generate potentially severe and life threatening soft-tissue and systemic infections [9]. Its ability to rapidly develop resistance to antibiotics creates a challenge to effectively treat *S. aureus* infections, especially those originating from methicillin resistant *S. aureus* (MRSA) strains [10,11]. Additionally, the propensity of *S. aureus* to grow and form biofilms adds another defense against antibiotic treatments [12].

In a previous study, we demonstrated that GRA exhibits bactericidal activity in vitro at 62.5 mg/L, but this activity was not observed in vivo [8]. However, sub-lethal treatment in vitro and topical treatment in vivo demonstrated that GRA reduced gene expression of key virulence factors and decreased the severity of skin infection in a mouse model of skin and soft-tissue infection [8]. GRA has also been shown to work synergistically with other antimicrobials [13]. The synergistic antibacterial effects observed in vitro and the ability to attenuate MRSA virulence in vivo through the targeting of non-essential genes present GRA as a potentially valuable supplementary treatment which, in conjunction with the employment of complementary antibiotic treatments, could help reduce or delay the development of antibiotic resistance. In the present study, we further explored the effects of sub-lethal doses of GRA, i.e., <62.5 mg/L, on the community-associated MRSA strain USA300 by evaluating its effects on cellular metabolism using ^1^H NMR metabolomics, cell–cell and cell–surface interactions, as well as its impact on the growth and viability of biofilm and free-floating planktonic MRSA cell cultures.

Metabolomics is a relatively new omics approach that aims to characterize the metabolomes, small molecule metabolite profiles, in organisms [14]. The advantage of metabolomics is that it provides close readouts of organism and cellular phenotypes. Metabolomics has been employed to between understand and characterize the molecular mechanisms underlying antimicrobial resistance and microorganisms’ adaptations to antibiotic selection pressures [15]. Two analytical techniques most commonly used in metabolomics research include mass spectrometry (MS) and nuclear magnetic resonance (NMR) spectroscopy [16,17]. NMR is particularly useful as it is quantitative, highly reproducible, and requires minimal sample preparations for the untargeted analysis of metabolite mixtures [18]. Herein, we have employed untargeted ^1^H NMR metabolomics to examine the impact of GRA treatment of the metabolic response and adaptation of MRSA.

## 2. Results

### 2.1. Increased Pigment Production upon GRA Treatment

**Figure 1:**
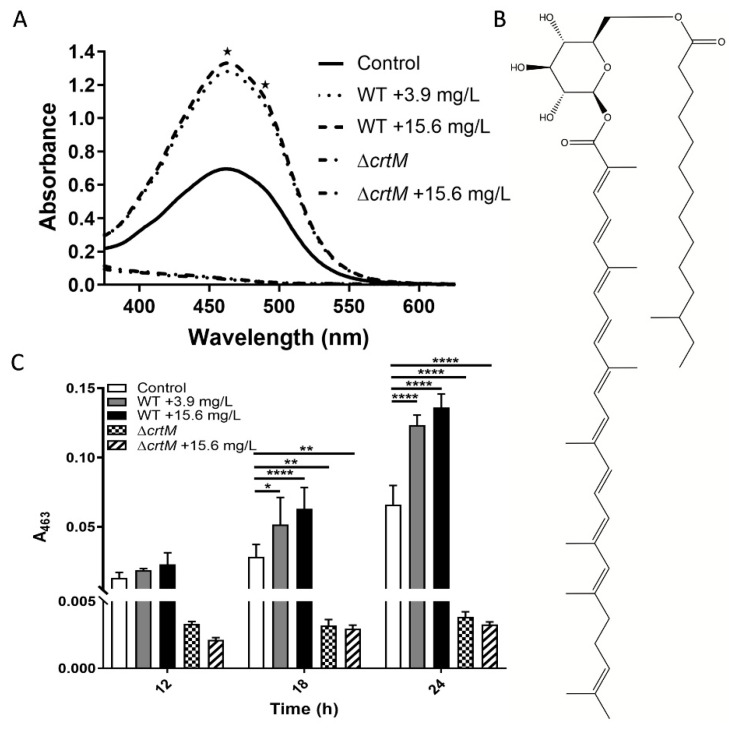
Staphyloxanthin (STX) Pigment Production post-Treatment with GRA. (**A**) Visible scans at 24 h reveal an overall increase in pigment with GRA treatment of WT MRSA bacteria and lack of pigment in the Δ*crtM* mutant strain that does not produce staphyloxanthin (STX). STX displays characteristic absorbance signals at 463 and 490 nm as indicated by the two asterisks. (**B**) Chemical structure of STX. (**C**) STX production was significantly higher at 18 and 24 h post-treatment with GRA in the WT MRSA strain compared to the untreated control, and absent in the Δ*crtM* mutant strain. Significance was established based on one-way ANOVA with Sidak post hoc test (* *p* ≤ 0.05, ** *p* ≤ 0.01, **** *p* ≤ 0.0001).

Treatment of wild-type (WT) planktonic MRSA cultures with sub-lethal doses of GRA induced a significant increase in pigmentation, compared to untreated controls. Visible wavelength scans of cellular extracts identified key signals at 463 nm and 490 nm (Figure 1A), which are characteristic of the well-characterized pigment staphyloxanthin [19]. The structure of STX (Figure 1B) illustrates how the hydrocarbon chains could allow STX to position itself in the lipid bilayer of the S. aureus cellular membrane, while the conjugated double bonds in the longer hydrocarbon chain can serve as a source of electrons to detoxify reactive oxygen species and allow STX to function as an antioxidant. The identity of the pigment was confirmed by assessing its potential production in the S. aureus transposon mutant Δ*crtM*, which is defective in the first enzyme of the biosynthetic pathway specific for staphyloxanthin [19,20]. Furthermore, monitoring the production of staphyloxanthin during planktonic cell growth confirmed that treatment with GRA stimulates increased production of staphyloxanthin relative to untreated MRSA bacteria at each timepoint (Figure 1C). The Δ*crtM* mutant strain produced no significant staphyloxanthin with or without GRA treatment (Figure 1C). STX production was monitored by measuring absorbance at 463 nm, and was found to be significantly higher at 18 and 24 h post-treatment with GRA in the WT MRSA strain, and significantly lower in the Δ*crtM* mutant strain relative to the untreated WT strain.

### 2.2. Metabolite Profiles of S. aureus Treated with GRA

**Figure 2:**
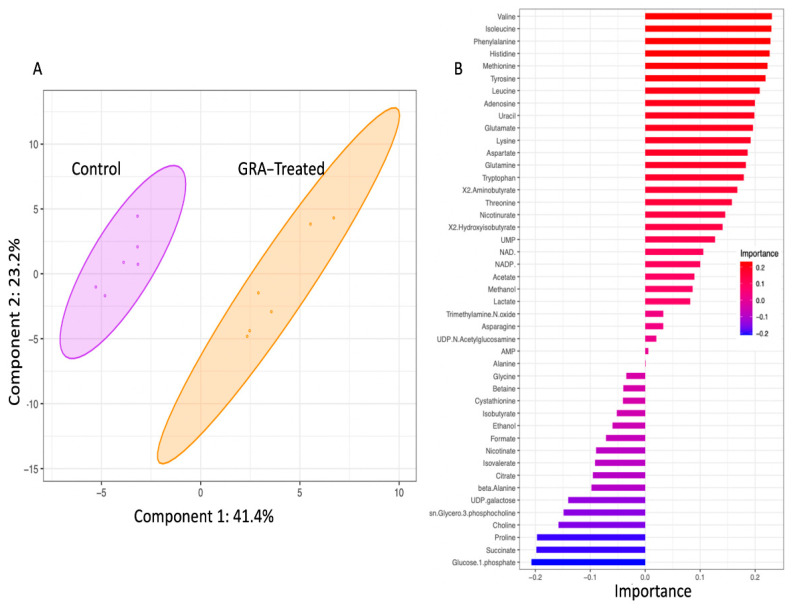
The 2D-PCA scores plot (**A**) and metabolites of importance associated with principal component 1 (PC1) of the PCA model (**B**). Multivariate statistical analysis of intracellular polar metabolite concentrations revealed distinct clustering of the untreated (purple) and 7.8 mg/L GRA treated (orange) bacteria. Shaded ellipses (shown in (**A**)) represent 95% confidence intervals. Metabolites of importance plot for component 1 (shown in (**B**)) indicate metabolite loading factors that contribute to the distinct separation of the two *S. aureus* cell culture groups along PC1 of the 2D PCA model; importance values of >0.10 or <−0.10 (as shown on the *x*-axis of plot (**B**)) are considered most noteworthy.

To generate insights into the effects of GRA on cellular metabolism, WT MRSA planktonic cell cultures were treated with 7.8 mg/L GRA for 1 h, at which point bacteria were harvested and intracellular metabolites extracted, followed by metabolite profiling using 1D ^1^H NMR spectroscopy. Multivariate statistical analysis based on principal component analysis (PCA) of differing metabolite concentrations revealed distinct clustering of the GRA-treated and untreated WT groups, and a clear separation of the two types of bacterial cultures (Figure 2A), with principal components 1 (PC1) and 2 (PC2) accounting for ~65% of the variance. Metabolites which contributed significantly to the group separation along Metabolites of interest included those with importance values >0.10 or <−0.10, and involved several amino acids: valine, isoleucine, phenylalanine, histidine, methionine, tyrosine, leucine, glutamate, lysine, aspartate, glutamine, tryptophan, threonine, and proline. Other important contributors to the separation between the GRA treated and control groups in the PCA model included the metabolites: adenosine, uracil, 2-aminobutyrate, nicotinurate, 2-hydroxyisobutyrate, UMP, NAD+, NADP+, glucose-1-phosphate, succinate, choline, sn-glycero-3-phosphocholine, UDP-galactose, β-alanine, and citrate.

**Figure 3:**
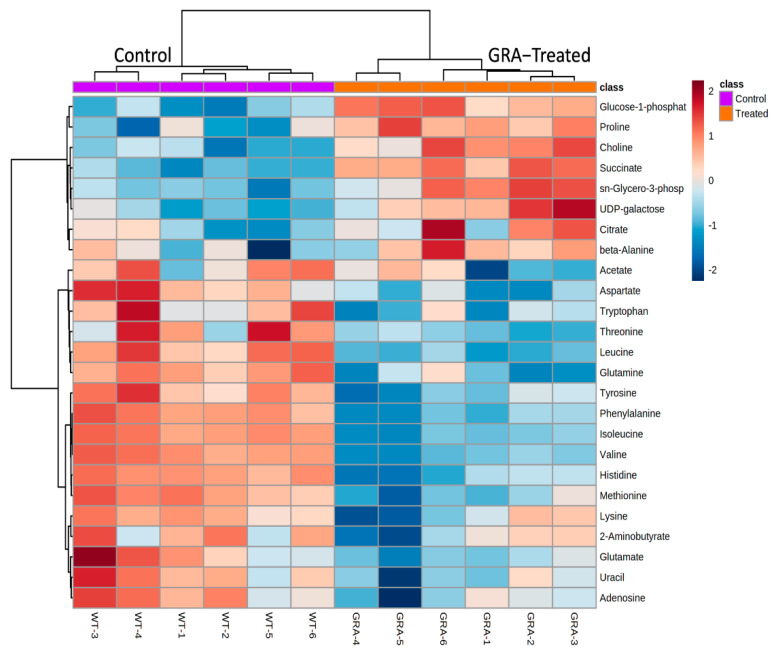
Hierarchical clustering analysis (HCA) and heatmap visualization of intracellular metabolite levels in the GRA-treated (orange) compared to the control (purple) bacterial cultures, with the top 25 metabolites with the most significant level differences that account for the separate clustering of the GRA-treated and untreated bacterial groups. Most notable are the higher levels of glucose-1-phosphate, proline, choline, succinate, sn-glycero-3-phosphocholine, UDP galactose, citrate, and beta-alanine in the GRA-treated cultures, while the levels of most of the other amino acids are higher in the untreated, MRSA control group.

**Figure 4:**
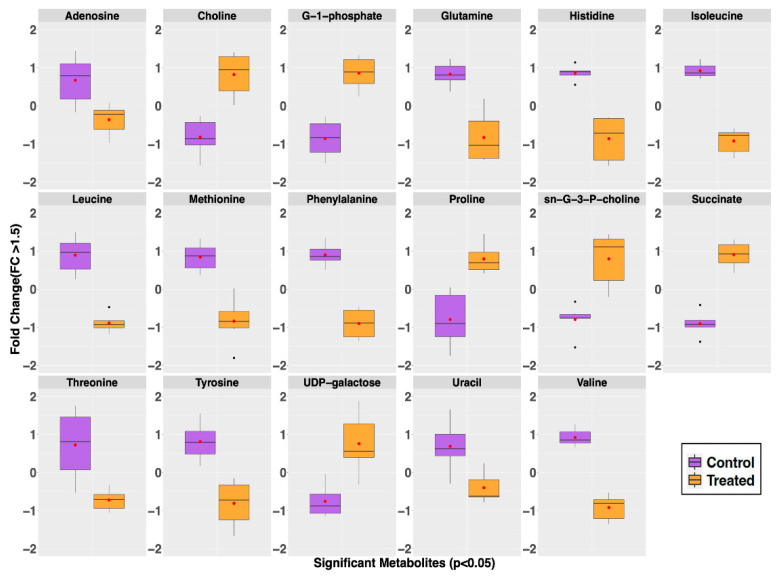
Intracellular metabolites whose level differences were significant (Fold change (FC) > 1.5 and *p*-value < 0.05) contributors for the separate classification of the GRA-treated (orange) from the untreated control (purple) groups. Whisker plots indicate ±1.5* interquartile range (IQR) observations; values > 1.5 and <3 *IQR are represented as small black dots with the mean represented by a single red dot. Most notable is the observation of higher levels of choline, proline, succinate, sn-glycero-3-phosphocholine (sn-G-3-P-choline), and UDP-galactose in the GRA-treated group, while the levels of adenosine, glutamine, histidine, isoleucine, leucine, methionine, phenylalanine, threonine, tyrosine, valine, and uracil are all lower in the GRA-treated group compared to the control group.

The separation of GRA treated from untreated MRSA cultures was also demonstrated using hierarchal clustering analysis of the characteristically distinct intracellular metabolite profiles of the two groups. Heatmap schematic representations (Figure 3 and Supplementary Figure S1) indicated higher levels of the following intracellular metabolites in the GRA-treated MRSA cultures: glucose-1-phosphate, proline, choline, succinate, sn-glycero-3-phosphocholine, UDP galactose, citrate, and beta-alanine; while almost all remaining amino acids and metabolites, such as uracil, adenosine, lactate, and acetate, were found in lower levels in the GRA-treated group compared to the untreated MRSA control cultures (Figure 3). In addition, the tricarboxylic acid (TCA) cycle intermediates succinate and citrate were found in slightly higher levels in the GRA-treated MRSA cultures, compared to the untreated control group (Figure 3 and Figure 4).

Volcano plot analysis identified 17 intracellular metabolites with a fold change >1.5 and *p* < 0.05. Most of these metabolites, including nine amino acids, were present in higher concentrations in the untreated MRSA control group (adenosine, glutamine, histidine, isoleucine, methionine, leucine, phenylalanine, threonine, tyrosine, valine, and uracil). A few were noticeably and significantly higher in concentrations in the GRA-treated group; these included choline, glucose-1-phosphate, proline, sn-glycero-3-phosphocholine, succinate, and UDP-galactose (Figure 4). A complete list of all metabolites identified and quantified, with corresponding means and standard deviations, is reported in the Supplementary Materials, Table S1.

### 2.3. Effect of GRA on Biofilms and Cell-Surface Adhesion

In addition to metabolomics investigations of the effect of GRA on MRSA planktonic cell cultures, the effects of GRA on established MRSA biofilms was also investigated. Since biofilms are inherently more resistant to antibacterial treatment, GRA concentrations corresponding to 1-2x the minimum inhibitory concentration (MIC) established for planktonic cell cultures were employed.

**Figure 5:**
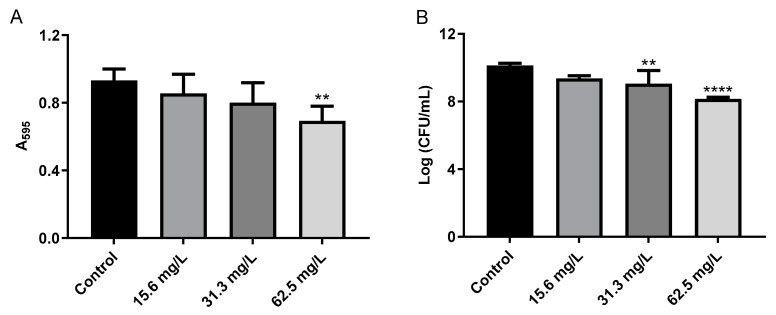
GRA disrupts bacterial cell-surface interactions. (**A**) Bacteria were cultured for 24 h in 24-well plates prior to treatment with 0, 15.6, 31.3, or 62.5 mg/L GRA. The extent of crystal violet staining was evaluated by measuring absorbance at 595 nm and was found to be significantly lower at 62.5 mg/L compared to control. This correlated with a loss in viable bacteria from the surface (**B**), indicating that GRA may enhance biofilm dispersal. The data shown in Figure 5 are the results from at least four experiments per treatment group. Significance was established based on one-way ANOVA (** *p* ≤ 0.01, **** *p* ≤ 0.0001).

Initially, GRA was found to have very little if any effect on cell viability following 24 h of treatment at 62.5 or 125 mg/mL as assessed by CFU counts of resulting cellular growth on tissue culture inserts (data not shown). However, we observed that, at the time of harvest, the GRA-treated biofilms did not adhere as tightly to the inserts as the untreated biofilms. This observation led us to further investigate the effects of GRA on MRSA cell-surface adhesion and cell–cell aggregation. In a cell-surface adhesion assay based on crystal violet staining of biofilms, treatment of MRSA biofilms with 62.5 mg/L GRA for 24 h resulted in a significant reduction in absorbance (A_595_), compared to the untreated MRSA biofilm control groups (Figure 5A). In addition, CFUs were significantly reduced following GRA treatment (Figure 5B). All together, these results thus suggested that treatment with GRA may enhance biofilm dispersal.

### 2.4. Bacterial Cell–Cell Aggregation

**Figure 6:**
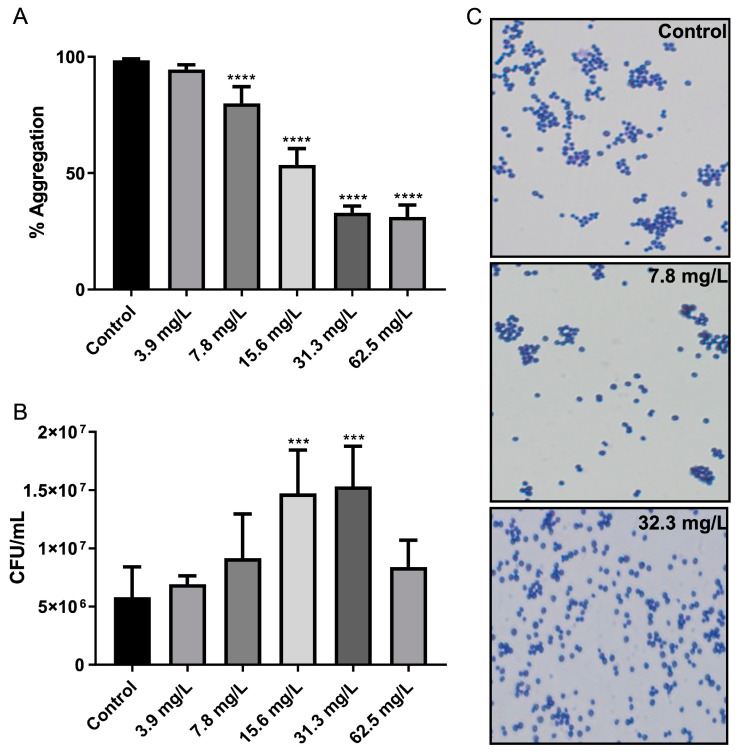
GRA disrupts bacterial cell–cell aggregation. Bacteria were cultured statically in test tubes in TSB containing 0.5% glucose and GRA for 24 h. Optical density measurements indicated significant disruption of cell–cell aggregation starting at 7.8 mg/L GRA compared to control (**A**). Reduction in cellular aggregation was associated with significant increases in CFU enumeration at GRA levels below the MIC (i.e., 62.5 mg/L), relative to controls (**B**). This observation was confirmed using microscopy at 100X agnification (**C**). Significance was established based on one-way ANOVA (*** *p* ≤ 0.001; **** *p* ≤ 0.0001).

Due to the observation of an effect of GRA on bacterial cell-surface adhesion within MRSA biofilms, the effect on cell–cell aggregation was also investigated using planktonic cell cultures. Treatment with GRA resulted in a dose-dependent effect on cell–cell aggregation, with higher GRA doses contributing to a greater disruption of cell aggregates (Figure 6A). Significant reductions in cellular aggregation were observed at concentrations as low as 7.8 mg/L GRA. CFU enumeration also revealed an increase in CFUs/mL at GRA concentrations below the minimal inhibitory concentration (MIC) (Figure 6B); this phenomenon is likely a result of reduced aggregation rather than an increase in cell number, as cellular aggregates tend to be accounted for as single CFUs. Gram-staining corroborated these findings with our qualitative observation of reduced cell–cell aggregation, as well as smaller overall size of aggregates, when present, compared to untreated MRSA control cell cultures (Figure 6C).

### 2.5. Treatment with GRA Reduces Expression of the Staphylococcal Alpha-Hemolysin (hla) Gene

**Figure 7:**
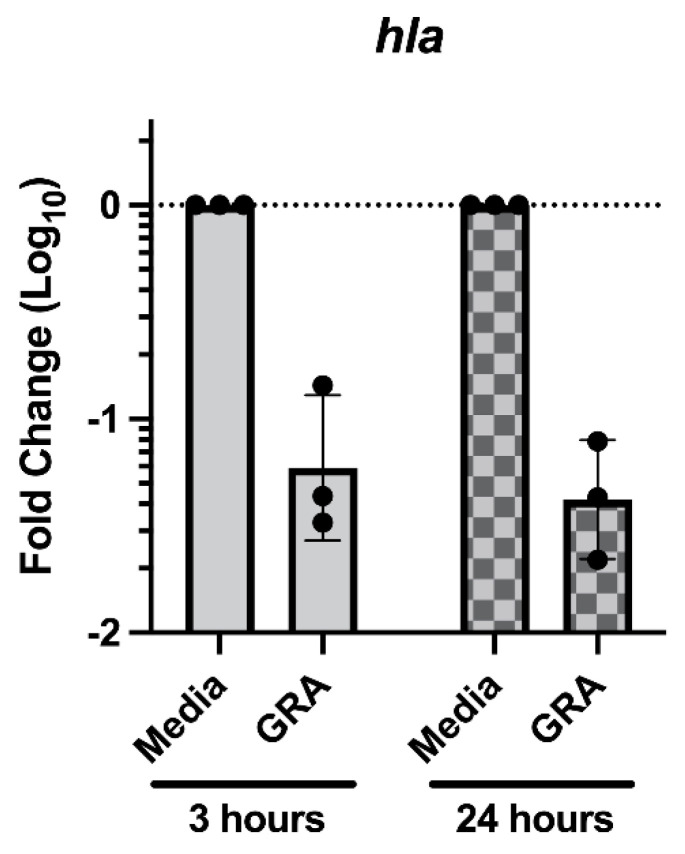
Treatment with GRA reduces biofilm *hla* transcript abundance. Static biofilms were grown for 24 h prior to media replacement with 62.5 mg/L GRA or DMSO control. RNA was isolated at 3- or 24 h post-treatment. Transcript abundance was evaluated using the 2^−ΔΔCt^ method, normalized to the expression of gyrase B (*gyrB*) housekeeping gene and calibrated to *hla* expression in media controls. Data displayed represent measurements from 3 biological replicates.

Previous studies demonstrated that treatment of *S. aureus* with GRA substantially decreased expression of the virulence alpha-hemolysin gene *hla* [8]. Recent studies have reported that *hla* expression is increased during biofilm growth on surfaces ranging from plastics to mucosal epithelia [21,22,23]. Taken together, we hypothesized that the observed reductions in cell–cell aggregations may result from decreased *hla* expression following GRA treatment. To evaluate this hypothesis, Taqman™ qRT-PCR was used to measure the abundance of *hla* transcripts in GRA treated biofilms at 3- and 24 h post GRA treatment. Consistent with previous studies, treatment of MRSA biofilms with GRA led to ~10-fold decrease in *hla* transcripts compared to *hla* transcript levels in untreated biofilms at both 3 and 24 h (Figure 7) [8].

Considering that biofilm matrices are complex environments composed of extracellular materials, including nucleic acid and proteins that can aid in cell aggregation [24], and that *S. aureus* displays an array of microbial surface components recognizing adhesive matrix molecules (MSCRAMMs) that can directly facilitate in cell adhesion [25,26], we next examined whether the observed biofilm dispersal effect of GRA is solely dependent on *hla* expression or a combination of increased enzyme and decreased MSCRAMM expression. For this purpose, transcript levels of nuclease (*nuc*), aureolysin (*aur*), fibronectin binding protein A (*fnbA*), and clumping factor A (*clfA*) were measured using qRT-PCR. These experiments revealed no significant increase in nuclease enzyme or decrease in gene expression of selected MSCRAMMs following GRA treatment of *S. aureus* biofilms when compared to untreated MRSA biofilm controls (data not shown). Collectively, these data support that decreased *hla* gene expression is a major contributing factor to the observed GRA induced disaggregation of MRSA biofilms, but additional studies are needed to identify other factors contributing to this phenotype.

## 3. Discussion

One of the more prominent effects of GRA is the enhanced production of staphyloxanthin (STX). STX is a signature carotenoid that imparts to *S. aureus* its characteristic golden color and is naturally expressed during stationary growth phase [27,28]. The production of STX is controlled by the *crt* operon, which comprises genes encoding the enzymes required for STX biosynthesis [19,20]. STX synthesis begins with condensation of two farnesyl diphosphates, products of the mevalonate pathway, a reaction catalyzed by dehydrosqualene synthase (*crtM*), which is followed by a series of steps leading to the final STX product. In addition to its natural occurrence in the life cycle of *S. aureus*, STX has been found to act both as an antioxidant [27,28,29] and a membrane stabilizer [30], and is involved in bacterial resistance mechanisms enabling cells to cope with environmental stressors, including exposure to antibiotics [30]. The numerous C=C bonds in the hydrophobic segment of STX serve as a rich source of electrons for the neutralization of reactive oxygen species, while also allowing STX to position itself within the lipid bilayer of the cellular membrane. We suspect that insertion of STX changes the physicochemical properties of the cell membrane, potentially altering membrane fluidity, which, together, may interfere with the cellular access of some antibiotics. Previous work has shown that increased STX levels are associated with an increased presence of branched chain fatty acid phospholipids in the membrane, which could potentially act to counterbalance an increased membrane rigidity that may be imparted by STX [31].

Our 1D ^1^H NMR metabolomics results indicated that the intracellular levels of all three branched chain amino acids (BCAAs, i.e., leucine, isoleucine, and valine) were significantly lower following GRA treatment, supporting the idea that these were used in cellular processes to shift membrane phospholipid composition [32,33]. Alternatively, lower BCAA levels could be an indication of alterations in *S. aureus* bacterial physiology as an adaptation response to GRA treatment [34]. Unexpectedly, choline levels remained significantly higher in GRA treated cells, despite the anticipated demand for choline in phospholipid synthesis, suggesting that choline uptake could be markedly increased and/or serving an additional purpose, such as osmoprotection and generation of glycine betaine [35,36]. Regardless of actual causes and effects, results from our study clearly demonstrate that GRA treatment leads to an increased production of STX in *S. aureus*, as well as a shift in central metabolism, which prompted further investigation.

Compared to untreated MRSA control cells, GRA imparted a significant shift in the metabolic landscape within an hour of treatment. Significant increases in intracellular levels of succinate and citrate suggest a potential dysregulation of the TCA cycle, which is also supported by the lower levels of several amino acids, indicating enhanced amino acid catabolism that may serve to replenish the TCA cycle intermediates [37]. Our findings are consistent with observations of others that investigated metabolome changes in *S. aureus* following treatment with the antibiotics, ampicillin and vancomycin [38]. Our results also suggest that catabolism of amino acids could be stimulated to enhance cellular production of ATP through more complex pathways [39]. This observation is consistent with GRA’s inhibition of DNA and protein synthesis which have previously been implicated in its mechanism of action [40]. TCA cycle activity and amino acid catabolism may also reflect the potential turnover of membrane lipids in response to the increased production of STX, as this process requires a significant amount of energy. Furthermore, previous research on other pentacyclic triterpenes (i.e., ursolic acid and oleanolic acid) has shown that incorporation of these molecules into cellular membranes changes membrane fluidity and leads to increased membrane rigidity [41].

Additional work by De Leon et al. with another Gram-positive bacterium, *Bacillus subtilis*, showed that triterpenoids induce cellular membrane damage as part of their mechanism of action [42]. Our results suggest that GRA may be working in a similar fashion, further potentiating its ability to alter the phospholipid composition of *S. aureus* cellular membranes, and ultimately driving ATP energy production and consumption of BCAAs. Such an increase in energy production could result in enhanced cellular oxidative stress and generation of reactive oxygen species which could stimulate STX production following GRA treatment, as observed in our study. Elevated choline levels could reflect *S. aureus* experiencing some degree of osmotic stress due to cellular membrane perturbations, which, in turn, would lead to recruitment of osmoprotectants, such as choline and/or proline (both of which are significantly elevated following GRA treatment) [12,43,44]. Although our metabolite profiling analysis provided valuable insights into metabolic changes associated with the activity of GRA against planktonic cells, we also wished to investigate the potential effects of GRA on MRSA biofilms which may be more relevant to clinical infections that are recalcitrant to current therapeutics.

Previous work on other triterpenes have found that their antibacterial activity is specific to Gram-positive microorganisms [41,42,45], including biofilm inhibition and reduction [46,47]. Despite GRA lacking bactericidal activity against MRSA biofilms, it is effective at reducing bacterial bioburden in two different biofilm models, suggesting that even at sub-lethal doses, GRA is potentially useful as a biofilm dispersal agent. Similar activity was observed against planktonic cultures treated with sub-MIC concentrations of GRA, which has led to significant reductions in micro-aggregation. Collectively, these results suggest that the cell dispersal effect observed in biofilms is at least in part associated with GRA interfering with cell–cell interactions. This interpretation of our findings is further supported by our previous work demonstrating that *hla* expression in planktonic cultures is down-regulated following treatment with sub-lethal doses of GRA [8], which we also observed in this study in GRA-treated biofilms. Work by others has identified alpha-toxin (*hla*) as a contributor to cell to cell interactions during biofilm formation and maturation [21]. The reduction in *hla* expression observed with GRA treatment in both biofilm and planktonic cell cultures could therefore explain, at least to some extent, the biofilm dispersal and reduced planktonic cell aggregation phenomena observed here with GRA treatment. Of note, the gene expression patterns seen here are consistent with a similar study wherein *S. aureus* aggregation was disrupted following treatment with sub-MICs levels of artesunate [48]. Artesunate and GRA share some structural similarity as terpene derivatives and are both reported to increase the susceptibility of MRSA strains to antibiotics [13,49]. Together, these data suggest GRA and artesunate may possess similar modes of action by disrupting *S. aureus* biofilms. Cell–cell aggregation in the planktonic cell cultures has also been linked to antibiotic resistance [50], but dispersal of these micro-aggregates is reported to reduce the lethality of lung infection [51]. Previous work has shown GRA to display synergistic effects with several aminoglycosides against planktonic cultures of MRSA, as well as with cetylpyridinium chloride, which has been used against Gram-positive *Streptococcus mutans* biofilms [47]. A more extensive investigation into how GRA impacts *S. aureus* gene expression and protein production involved in aggregation and whether this is due to GRA altering cellular membrane fluidity is warranted.

Overall, our study supports the use of GRA as a potentially effective antibacterial against MRSA planktonic and biofilm cultures via its significant impact on cell–cell aggregation. Although disruption of biofilms is controversial as to whether it could pose a greater risk to the host [52,53], GRA, in combination with other appropriate treatment regimens, could be useful as a therapeutic agent to restore efficacy to antimicrobials that are no longer considered effective due to the rapid development of antibiotic resistance when used alone. In addition, the physiological effects of GRA imparted at sub-MIC levels could serve to minimize future development of resistance against GRA, further contributing to mitigating the development of antimicrobial resistance in *S. aureus*.

## 4. Materials and Methods

### 4.1. Bacterial Strains

The community-associated MRSA strain LAC (Los Angeles County, CA, USA), pulsed-field gel-electrophoresis type USA300 (wild-type, WT) [54,55,56] and the JE2 transposon mutant USA300 NE1444 (∆*crtM*) acquired from the Network on Antimicrobial Resistance in Staphylococcus aureus were used in this study. Growth conditions (i.e., media, temperature, shaking speed) are described in assays below

### 4.2. Stock Preparation of 18-β-Glycyrrhetinic Acid

All stocks were prepared in 100% dimethyl sulfoxide (DMSO) at 25 mg/mL and stored at −20 °C until needed. Control samples were dosed with equivalent amounts of DMSO based on level of treatment.

### 4.3. Pigmentation Assay

WT and ∆*crtM* strains were cultured to mid-log phase (OD_600_ ~ 0.35) in cation-adjusted Mueller–Hinton Broth (MHB) at 37 °C with shaking at 250 rpm. Bacteria were diluted to approximately 5.5 × 10^5^ CFU/mL in fresh MHB containing either 3.9 or 15.6 mg/L GRA (controls were treated with an equivalent of DMSO). Cultures were incubated as above and sampled at 12, 18, and 24 h. At each time point, bacteria were harvested, washed with 1X PBS, and then pelleted. After removing PBS, pellets were frozen at −80 °C for at least 12 h before extracting pigment. To extract the pigment, frozen pellets were thawed and resuspended in 1 mL of 100% methanol, followed by incubation at 55 °C for 5–10 min in a water bath. Samples were vortexed before and after incubation, as well as 1-2x during incubation. Following incubation, debris were pelleted and supernatants containing the pigment were collected for spectral analysis. Supernatants were scanned from 300–700 nm using a Thermo Scientific Genesys 10S UV-Vis Spectrophotometer.

### 4.4. Metabolic Sample of WT Untreated and Treated with GRA

WT bacteria were grown to mid-log phase in TSB (50 mL in 150 mL flask) at 37 °C with shaking at 250 rpm. At this point, bacteria were diluted into fresh media containing 7.8 mg/L GRA or an equivalent amount of DMSO to a final concentration of ~2.3 × 10^7^ CFU/mL. A sub-lethal dose was chosen to mitigate metabolic changes associated with bacterial cell death. Cultures were further incubated with shaking at 250 rpm for 1 h at 37 °C, harvested by centrifugation, and the resulting pellets washed once with ice-cold 1X PBS before freezing at −80 °C. Pellets for all samples were resuspended in 1 mL of 2:1 methanol:chloroform to extract intracellular metabolites [57]. At this and all subsequent steps, samples, and reagents were kept on ice.

Following resuspension in methanol:chloroform, samples were transferred to a 2 mL tube containing 0.7 g of 0.1 mm silica beads. Tubes were placed in an MP Biomedicals FastPrep-24^TM^ 5G and lysed for 2 × 40 s cycles at a speed of 6.0, keeping tubes on ice between cycles. Following lysis, the resulting slurry was adjusted to a ratio of 1.0:1.0:0.5 methanol:chloroform:water, briefly vortexed, and then centrifuged for 10 min at 14,000 rpm at 4 °C to separate the aqueous and non-polar phases. The aqueous fraction (top phase) was removed gently with a pipette, and subsequently dried using a vacuum centrifuge overnight with no heat. Resulting metabolite mixtures were stored at −80 °C until NMR analysis. Samples collected represented 6 biological replicates.

### 4.5. 1D ^1^H NMR Analysis

For NMR experiments, samples were resuspended in 750 μL of NMR buffer containing 10 mM NaH_2_PO_4_/Na_2_HPO_4_, 0.4 mM imidazole (pH indicator), and 0.25 mM 4,4-dimethyl-4-silapentane-1-sulfonic acid (DSS, ^1^H chemical shift reference indicator) in 10% D_2_O, pH 7.0. Samples were spun at 13,000 rpm for 2 min to remove any debris and then 700 μL was transferred to a 5 mm Wilmad NMR tube. One-dimensional (1D) ^1^H NMR spectra were recorded at 298 K on a Bruker 600-MHz AVANCE III solution NMR spectrometer equipped with a SampleJet automatic sample loading system, a 5 mm triple resonance liquid-helium-cooled TCI probe, and the Topspin software (Bruker version 3.2). One-dimensional ^1^H NMR experiments were performed using the Bruker *zgesgp* pulse sequence with 256 scans, a ^1^H spectral window of 9600 Hz, 32K data points, and a dwell time interval of 52 μs amounting to an acquisition time of 1.7 s, and a 1 s relaxation recovery delay between acquisitions [58].

The resulting spectra were processed using Topspin 3.2, and NMR spectral feature analyses and metabolite identifications were conducted using the Chenomx NMR Suite software (version 8.0) as described by Fuchs et al. [59]. Following spectral phasing and baseline correction, a line broadening function of no more than 0.5 Hz was employed, as needed, following Chenomx protocols and reported NMR metabolomics methods [60,61]. ^1^H Chemical shifts were referenced to DSS whose most upfield NMR signal was set at 0.0 ppm, and the NMR signal from imidazole was used to correct for small chemical shift changes due to slight pH variations. NMR signals were quantified from relative signal intensity, and annotated by matching chemical shift and spectral splitting patterns to those of reference spectra accessible through the Chenomx 600 MHz (^1^H Larmor frequency) spectral database of small molecule metabolites [59,60]. Using the Chenomx software, complex NMR spectral patterns obtained from the 1D ^1^H NMR spectra of resulting metabolite mixtures were deconvoluted and used for identification and quantification of 45 distinct metabolites from the two different treatment groups (GRA treated and control).

Validation of annotated metabolite IDs was accomplished using 2D ^1^H-^1^H (TOCSY) NMR or by spiking samples with pure metabolite standards, if necessary. Acquisition of 2D ^1^H-^1^H TOCSY spectra employed the Bruker-supplied ‘mlevphpr.2/mlevgpph19′ pulse sequences, and following experimental parameters: 256 t1 points; 2048 t2 data points, 2 s relaxation delay, 32 scans per t1 interval, ^1^H spectral window of 6602.11 Hz, and 80 ms TOCSY spin lock mixing period. 2D ^1^H-^1^H TOCSY spectra were processed and analyzed using Topspin software.

A concentration table (in µM) of 45 unambiguously identified metabolites were then exported from the Chenomx software as a .csv (comma separated values) file which was used for multivariate and univariate statistical analysis.

### 4.6. NMR Statistical Analysis

Metabolic concentrations were established relative to DSS and further normalized to viable cell counts, i.e., colony forming units or CFUs. Statistical analyses, including PCA, HCA, and Volcano plot analyses, were performed using the open-source software MetaboAnalyst and its MetaboAnalystR package [62]. As a first step, metabolite concentration datasets were log-transformed to ensure a Gaussian distribution of the data and auto-scaled (i.e., mean centered and divided by the standard deviation) prior to statistical analysis, including 2D principal component analysis (2D-PCA) and hierarchical clustering analysis (HCA). HCA was conducted in MetaboAnalyst using a Euclidean distance measure and Ward clustering algorithm. All 45 metabolites, as well as the top 25 most discriminating metabolites that were identified and quantified, were used to generate heatmap graphical representations of the data, and to characterize the metabolite level patterns that discriminate and separate the GRA treated from untreated MRSA cell cultures.

### 4.7. Biofilm Activity Assay

Activity against MRSA biofilms was assayed using a tissue cell culture plate method modified from Kirker et al. [63]. Bacteria were grown planktonically in TSB supplemented with 0.5% glucose for 18 h at 37 °C with shaking at 250 rpm. Tissue culture inserts were inoculated with five 10 μL droplets and allowed to set for 20 min before adding 1.5 mL of TSB to each well below the insert. The insert rested on top of the media to promote biofilm formation on the insert rather than planktonic growth in the well. Plates were incubated for 48 h, refreshing the media at 24 h, before treatment with 62.5 or 125 mg/L GRA (or DMSO equivalent for controls) in TSB. Biofilms were harvested 24 h post-treatment and plated for CFUs on LB agar. Agar plates were incubated overnight at 37 °C and CFUs enumerated the next day.

### 4.8. Cell-Surface Adhesion and Disruption

Biofilm adherence was assessed using a modified assay from Cassat et al. [64]. Bacteria were cultured planktonically in TSB glucose for 18 h at 37 °C with shaking at 250 rpm and then diluted 1:200 into fresh media. A 24-well cell culture plate was inoculated with 1 mL aliquots of this diluted culture and incubated statically for 24 h at 37 °C. At 24 h, the old media was carefully removed and replaced with fresh media containing GRA (or DMSO equivalent for controls) at a range of concentrations. Following an additional 24 h of incubation, biofilms were washed with 1X PBS and then either were harvested with 1 mL 1X PBS for counting viability or stained with crystal violet to measure the degree of biofilm adherence. For CFU viability, each cell suspension collected from the inserts was diluted as needed and plated on LB agar. Plated bacteria were incubated overnight at 37 °C and CFUs enumerated the next day.

Biofilms were stained with 1% *w*/*v* crystal violet for 15 min. Excess stain was then removed, and biofilms were gently rinsed 3× with 200 μL H_2_O. Stained biofilms were dried overnight at room temperature followed by de-staining with 30% acetic acid for 15 min. The extracted crystal violet was measured at an absorbance of 595 nm [64,65].

### 4.9. Bacteria Cell–Cell Aggregation and Microscopy

Bacterial cell–cell aggregation was assessed using a slightly modified protocol from Geoghegan et al. [66]. Planktonic cultures of WT bacteria were initially cultured at 37 °C with shaking at 250 rpm. At 18 h, cells were pelleted, resuspended in an equal volume of fresh TSB, and then diluted 1:3 into 10 mL tubes. Suspensions were treated with GRA (or DMSO for control samples) and test tubes were incubated without shaking for 24 h at 37 °C. Following incubation, OD_600_ was recorded for the top 1 mL of each sample. Samples were then vortexed vigorously to resuspend the cells, followed by a second measurement of the OD_600_. Percent aggregation was calculated as follows:

(1) $$\%\;\mathrm{Aggregation}=\frac{\left(\mathrm{Post}\text{-}\mathrm{vortexed}\;\mathrm{OD}600)-(\mathrm{Pre}\text{-}\mathrm{vortexed}\;\mathrm{OD}600\right)}{\left(\mathrm{Post}\text{-}\mathrm{vortexed}\;\mathrm{OD}600\right)}*100$$

Bacterial cell–cell aggregation was confirmed by Gram-staining and microscopy at 100X magnification of individual treatments or control. Prior to staining, samples were diluted 1:10 in 1X PBS and 10 μL was pipetted onto a glass slide. Slides were flame-dried over a Bunsen burner and allowed to cool before Gram-staining.

### 4.10. RNA Extraction and Quantitation

To evaluate transcriptional level changes in biofilms due to treatment with GRA, overnight planktonic cultures of WT cells were diluted 1:200 in fresh TSB, followed by transferring 1 mL each well of a 24-well cell culture plate. Plates were incubated statically for 24 h at 37 °C to establish biofilms, followed by gently replacing the media with fresh TSB with 62.5 mg/L GRA or equivalent DMSO control. At 3 h and 24 h post-treatment, biofilms were resuspended in growth media, transferred to microcentrifuge tubes, and samples pelleted by spinning at 10,000 rpm for 2 min. Supernatants were removed and cell pellets were resuspended in 400 µL RLT + beta-mercaptoethanol (Qiagen, Germantown, MD, USA). Samples were immediately stored in −80 °C until further analysis. All samples were generated in biological triplicates. RNA was purified using an adaptation of the RNeasy Kit (Qiagen, Germantown, MD, USA) manufacturer’s protocol as described by Voyich et al. [67]. TaqMan RT-PCR was performed using the primer and probe sets detailed in the Supplementary Materials section, Table S2. Transcript abundance was evaluated using the 2^−ΔΔCt^ method and normalized to the expression of the housekeeping gene gyrase B (*gyrB*) as previously described [8]. Data shown are relative to media controls at corresponding time points.

## Data Availability

All data are available as supplementary materials or upon request to the authors.

## Supplementary Materials

The following supporting information can be downloaded at: https://www.mdpi.com/article/10.3390/antibiotics11060781/s1, Figure S1: Hierarchical clustering analysis (HCA) and heatmap visualization of intracellular metabolite levels in the GRA-treated (orange) compared to the control (purple) bacterial cultures, including all metabolites that were identified and quantified. Table S1: List of metabolites and their mean concentrations (in [(μmoles/CFU) × 10^−14^], measured in the GRA treated and the control *S. aureus* cell culture groups. Table S2: Table of primers and probe sequences that were used in the TaqMan RT-PCR experiments.
