# Supplementary material for: 18β-Glycyrrhetinic Acid Induces Metabolic Changes and Reduces Staphylococcus aureus Bacterial Cell-to-Cell Interactions
Source: Antibiotics (Basel). 2022 Jun 8;11(6):781. doi: 10.3390/antibiotics11060781 (PMC9220049; doi:10.3390/antibiotics11060781)
Supplement: Supplementary file 1 [file antibiotics-11-00781-s001.zip › Supplementary_Final.pdf]

Supplementary Figure S1: Hierarchical clustering analysis (HCA) and heatmap visualization of intracellular metabolite levels in the GRA-treated (orange) compared to the control (purple) MRSA cell cultures, with all metabolites identified and quantified displayed.

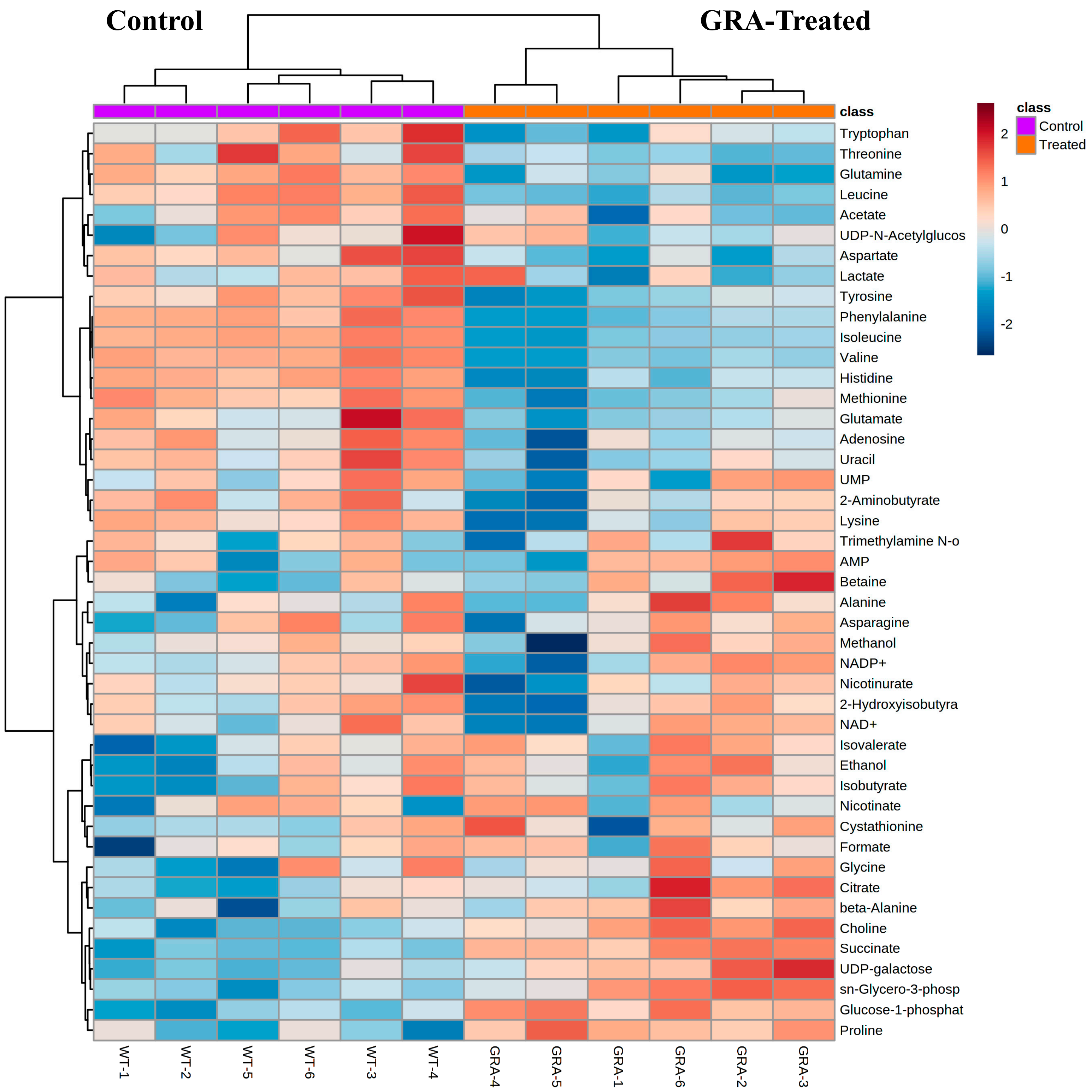

Supplementary Table S1: List of intracellular metabolites and mean concentrations, as measured by ^{1}H NMR, for the GRA-treated and control *S. aureus* cell cultures; Metabolite concentrations are reported as the mean concentration ± one standard deviation [(µmoles/CFU)*10^{-14}].

	**Control**		**GRA Treated**	
	Mean	Std. Dev.	Mean	Std. Dev.
2-Aminobutyrate	15.28	6.78	7.79	4.37
2-Hydroxyisobutyrate	3.69	0.38	3.34	0.63
AMP	6.87	2.37	7.90	2.59
Acetate	91.59	18.24	70.46	16.85
Adenosine	4.56	1.71	2.03	0.83
Alanine	50.25	4.27	52.16	5.26
Asparagine	17.96	3.68	17.76	3.11
Aspartate	375.43	48.23	283.19	27.98
Betaine	505.58	48.54	568.88	86.09
Choline	4.39	0.57	6.89	1.06
Citrate	11.67	1.02	13.60	1.81
Cystathionine	14.09	1.64	15.09	2.96
Ethanol	1.76	0.50	2.07	0.47
Formate	1.42	0.28	1.58	0.24
Glucose-1-phosphate	1.63	0.43	4.16	0.95
Glutamate	196.54	23.12	163.66	9.33
Glutamine	54.61	11.09	19.50	9.26
Glycine	46.60	7.58	49.56	5.14
Histidine	3.17	0.37	1.16	0.40
Isobutyrate	0.48	0.12	0.54	0.09
Isoleucine	18.43	1.82	7.45	1.19
Isovalerate	0.83	0.18	0.98	0.16
Lactate	104.05	19.40	86.04	26.84
Leucine	33.92	7.11	15.18	1.68
Lysine	12.70	1.43	9.01	2.85
Methanol	2.22	0.32	2.17	0.87
Methionine	18.84	2.84	9.57	2.33
NAD+	23.19	3.32	21.89	4.72
NADP+	2.69	0.29	2.59	0.58
Nicotinate	1.13	0.82	1.44	0.94
Nicotinurate	1.11	0.70	0.72	0.46
Phenylalanine	5.34	0.77	2.25	0.43
Proline	41.20	10.43	70.38	10.00
Succinate	3.71	0.52	8.36	1.28
Threonine	16.10	9.91	4.62	0.96
Trimethylamine N-oxide	256.89	26.90	260.95	43.37
Tryptophan	1.56	0.33	1.09	0.19
Tyrosine	4.02	0.73	2.26	0.48
UDP-N-Acetylglucosamine	2.69	1.93	1.99	0.70
UDP-galactose	1.88	0.36	3.71	1.30
UMP	2.90	0.63	2.49	0.84
Uracil	2.27	0.50	1.45	0.35
Valine	31.54	3.34	13.79	2.05
sn-Glycero-3-phosphocholine	16.58	2.86	36.30	11.04
beta-Alanine	5.89	1.52	7.94	1.69

Supplementary Table S2. Primers & Probes for qRT-PCR.

**Primers and Probes**	**Sequence**
clfA forward	5' - AGGTTCTGGTGACGGTATCGA - 3'
clfA reverse	5' - TCAATTTCACCAGGCTCATCAG - 3'
clfA probe	5' - AAACCAGTTGTTCCTGAAC - 3'
fnbA forward	5' - CGTCAAACGCAACACAAGTAA - 3'
fnba reverse	5' - CGCTTCTTCCTTAACTACCTCTT - 3'
fnba probe	5' - AAGCACCACAAACTGCACAACCAG - 3'
gyrB forward	5’ - CAAATGATCACAGCTTTGGTACAG - 3’
gyrb reverse	5’ - CGGCATCAGTCATAATGACGAT - 3’
gyrb probe	5’ - AATCGGTGGCGACTTTGATCTAGCGAAAG - 3’
hla forward	5’ - CAACAACACTATTGCTAGGTTCCATATT -3’
hla reverse	5’ - CCTGTTTTTACTGTAGTATTGCTTCCA -3’
hla probe	5’ - ATGAATCCTGTCGCTAATGCCGCAGA -3’
nuc forward	5' - ATATGGACGTGGCTTAGCGT - 3'
nuc reverse	5' - TGAATCAGCGTTG TCTTCGCTCCA - 3'
nuc probe	5' - ACGAAGCTTTAGTTCGTCAAGGCTTGGC - 3'
